# Motivation in adolescents and caretakers undergoing a weight management program – longitudinal data from the cluster-randomized, motivation-focused STARKIDS trial

**DOI:** 10.1186/s12916-026-05332-7

**Published:** 2026-11-04

**Authors:** Anne Herschbach, Florian Junne, Rebecca Erschens, Stefan Ehehalt, Johann Jacoby, Peter Martus, Isabelle Mack, Alisa Weiland, Inga Krauß, Constanze Greule, Gorden Sudeck, Guido Zurstiege, Annalena Wels, Sophia Helen Adam, Andreas Hoell, Wolfgang Bethge, Monika Ums, Torben Sammet, Maren Reyer, Olaf Schliesing, Stephan Zipfel, Caterina Gawrilow, Katrin Elisabeth Giel, Katrin Ziser

**Affiliations:** 1 Department of Psychosomatic Medicine and Psychotherapy, University Hospital Tuebingen, Tuebingen, Germany; 2 Department of Psychosomatic Medicine and Psychotherapy, Otto von Guericke University Magdeburg, Magdeburg, Germany; 3 Public Health Department of Stuttgart, Stuttgart, Germany; 4 Institute of Clinical Epidemiology and Applied Biometry, Medical Faculty, University of Tuebingen, Tuebingen, Germany; 5 Department of Sports Medicine, Medical University Hospital Tuebingen, Tuebingen, Germany; 6 Interfaculty Research Institute for Sport and Physical Activity, University of Tuebingen, Tuebingen, Germany; 7 Institute of Sports Science, University of Tuebingen, Tuebingen, Germany; 8 Insitute of Media Studies, University of Tuebingen, Tuebingen, Germany; 9 Clinic for Psychiatry and Psychotherapy, Medical Faculty Mannheim, Central Institute of Mental Health, University of Heidelberg, Mannheim, Germany; 10 Center for Clinical Trials, Medical Faculty, University of Tuebingen, Tuebingen, Germany; 11 Ministry for Social Affairs, Health and Integration Baden-WürttembergDepartment Health Department, Stuttgart, Germany; 12 Data Medico GmbH, Heidelberg, Germany; 13 German Centre for Mental Health (DZPG), Partner site Tuebingen, Tuebingen, Germany; 14 Department of Psychology, University of Tuebingen, Tuebingen, Germany

**Keywords:** Overweight, Obesity, Children, Adolescents, Motivation, Weight management, Weight loss, Transtheoretical model

## Abstract

**Background:**

Motivation towards behavior change is an important determinant of long-term weight management. Previous research demonstrates that children’s and adolescents’ motivation can be increased through participation in weight management programs. However, little is known about motivational changes in caretakers of children and adolescents with overweight and obesity despite their recognized role as “agents of change” in pediatric weight management. The study aims to compare motivational changes in caretakers and adolescents between a group receiving a motivation-focused weight management program and a control group. Furthermore, predictors for motivational changes are unknown.

**Methods:**

This study is a secondary analysis of the cluster-randomized STARKIDS trial. The intervention group received an 18-month motivation-focused weight management program, the control group received optimized treatment-as-usual. Motivation was assessed based on Transtheoretical Model, using the University of Rhode Island Change Assessment-Short, as well as the related parent-perspective version. Each of these instruments contains four subscales: Precontemplation, Contemplation, Action, and Maintenance. Linear mixed models with multiply imputed data were employed to evaluate changes within the subscales between pre- to post-intervention measurements and to compare these changes between the intervention and control group. A multiple linear regression was subsequently used to identify predictors of motivational change.

**Results:**

Caretakers in the intervention group showed significantly greater changes on Maintenance subscale compared to caretakers in the control group at both T0-T1 and T0-T2 and trends for these changes on Action subscale. No group differences were found in adolescents. Among caregivers in the intervention group, multiple regression analysis for T0-T1 changes on Action subscale identified parent-reported adolescents’ obesity-related quality of life as significant negative predictor for an increase on Action stage.

**Conclusions:**

This motivation-focused weight management program produced motivational gains at the Maintenance stage, supporting sustained behavioral change. Baseline obesity-related quality of life predicted motivational change, suggesting its value as a clinical marker for tailoring interventional approaches. Future research should explore distinct motivational trajectories to inform more individualized pediatric weight management strategies.

**Trial registration:**

Preregistered in the German Clinical Trials Register (DRKS) DRKS000228 13 (acknowledged primary register of the World Health Organization) on 27 November 2020 (Universal Trial Number U111112549536).

**Supplementary Information:**

The online version contains supplementary material available at https://doi.org/10.1186/s12916-026-05332-7.

## Background

A 2025 UNICEF report stated that the global prevalence of overweight (OW) in children has recently surpassed that of underweight [1]. Indeed, the prevalence of pediatric OW and obesity (OB) has substantially increased in recent decades [2]. This is cause for concern because pediatric OW and OB are associated with serious secondary diseases and consequences including somatic diseases [3, 4], mental health disorders [5], and psychosocial complaints [6]. Furthermore, pediatric OB frequently persists into adulthood [7] and is associated with premature mortality [8]. Lastly, the economic consequences of OB and OW are immense [9]. Taken together, these findings emphasize the urgent need for prevention and early treatment. Current clinical guidelines recommend multicomponent weight management programs for children and adolescents, combining interventions targeting nutrition, physical activities and behavioral change [10–12]. Additionally, weight management programs for children and adolescents should be directed to the whole family and should foster motivation (e.g. 10,11).

Weight management is a long-term process that does not end when participation in a weight management program is finished. Following initial weight reduction, participants of weight management interventions often regain weight [13]. In order to help children and adolescents to reach a healthy weight, we need to find ways to support the long term maintenance of physical activity and healthy diet into daily routines [14]. This responsibility does not lie with the individual alone: behavior is embedded in family, social, environmental and policy contexts. Accordingly, the Behavior Change Wheel [15] situates motivation alongside capability and opportunity within a broader system that also includes environmental and policy-level levers. Within this system, motivation towards behavior change remains a key modifiable component at the level of individual and family-based interventions and is therefore the focus of the present study. Motivation towards behavior change means a superordinate form of motivation regarding behavior changes that lead to a healthy weight (e.g. changing eating and physical activity behavior, participating in a weight management program etc.). Previous research shows the important role of motivation in weight management [16, 17]: Motivation can have effects on attrition of weight management programs and on the effectiveness of these programs [18, 19]. In contrast, participation in weight management programs can also effect motivation: A systematic review shows that motivation for life-style adaptations in adolescents was higher after participation in weight management than before [16].

In pediatric weight management, it is essential to consider the motivation of both the affected child / adolescent and the accompanying parent or family, since a dyadic or systemic behavior change is required [20–22]. However, a systematic review of the existing literature shows that most studies focus on the motivation of children or adolescents, with comparatively few addressing the motivation of parents or caretakers [16]. This is particularly noteworthy given the well-established role of parents as relevant “agents of change” in pediatric weight management [23]. Pediatric weight management programs aim to change children’s and adolescents’ weight, however parental support is strongly needed. Parents may have to change the surroundings of the children and adolescents and have to change e.g. own cooking habits. In addition, they need to support their children. Therefore, there is a research gap regarding the change in motivation of parents involved in weight management programs [16, 22].

**Fig. 1:**
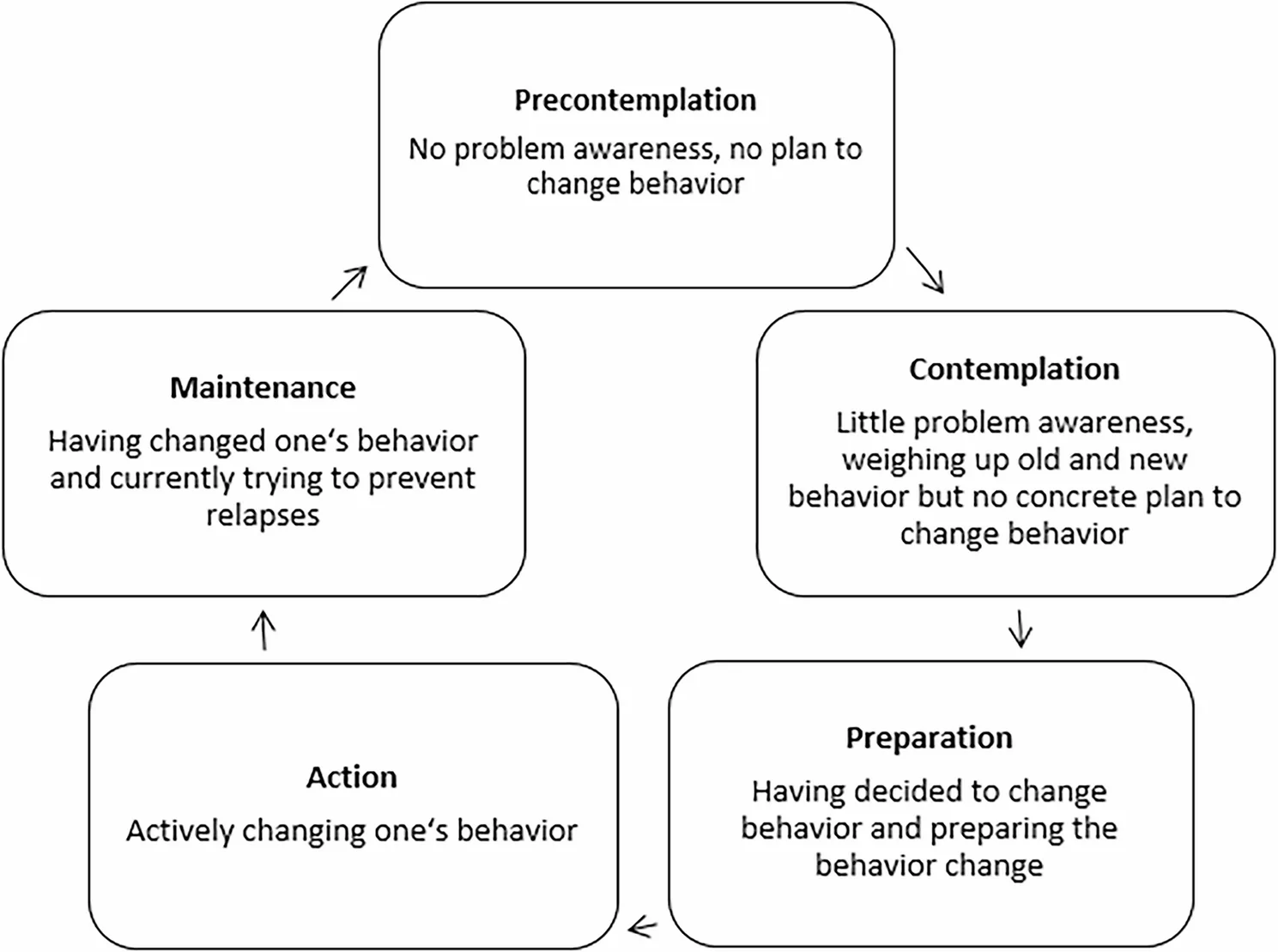
Five SoC according to Prochaska and DiClemente [24]

Motivation regarding behavior change in pediatric weight management is operationalized and measured in various ways which complicates comparison across studies (for an overview, see 16). Common ways to assess motivation to change is the Transtheoretical Modell (TTM) by Prochaska and DiClemente [24] and the Self-determination theory (SDT) by Deci and Ryan [25]. Both describe how human change their behaviors. The TTM states that this process of change is accompanied by a sense of ambivalence, whereby the individual is uncertain as to whether the new behavior (in this case, a healthy lifestyle) is preferable to the old behavior. According to the TTM, behavioral change unfolds across six stages of change (SoC): Precontemplation, Contemplation, Preparation, Action, Maintenance, and Termination. These stages are illustrated in Fig. 1. Weight management interventions aim to specifically address the stages of Action and Maintenance since they are associated with actively changing one’s behavior and sustained efforts to prevent relapses which could led to successful weight management. The TTM has been criticized for insufficient empirical evidence and that application to SoC may be arbitrary [26]. However, Prochaska et al. [27] expanded the model by describing progression on the SoC as a circular process which can include relapse, regression, omission, and renewed initiation. The addition of relapses to the TTM makes it suitable for research on pediatric OB, as attrition is common [28]. Furthermore, the TTM reflects behavior change as a long-term process which makes it appropriate for weight management. In addition, the TTM takes ambivalence and different levels thereof into account, which is commonly seen in weight management [29]. Consequently, the TTM was chosen as the most suitable theoretical background for the present study. Within the TTM, individuals typically endorse attitudes from several stages simultaneously, and the continuous subscale scores capture the relative weight of these attitudes. Therefore, in the present study, increases in motivation do not refer to transitions between discrete SoC, but to changes in the degree of endorsement of stage-specific attitudes. That is, to a shift in the motivational profile towards later SoC (higher Action and Maintenance scores and/or lower Precontemplation scores). An increase within a subscale can therefore be understood as a consolidation of the corresponding motivational orientation. Rising Maintenance scores, for instance, indicate that relapse prevention and the stabilization of already initiated changes become increasingly salient, even when no categorical transition occurs. Previous research on other disorders (e.g. substance abuse) has found an association between higher scores on Action and Maintenance stages and improved clinical outcomes [30–32]. Such within-stage consolidation is clinically meaningful in pediatric OB, as it reflects how firmly behavioral changes are anchored in everyday family routines and is particularly relevant given the high rates of weight regain after interventions.

In summary, existing evidence indicates that motivation towards behavior change in children and adolescents increases during participation in weight management programs [16]. However, data on how parents’ motivation evolves and the impact of a motivation-focused treatment on this population remain insufficient. Furthermore, the factors that drive motivational change in this context are poorly understood. While certain variables associated with baseline motivation in pediatric weight management have been identified (e.g. higher income, less parental OW, [33, 34]), the determinants of motivational change over time remain unclear. A preceding study of baseline data from this sample revealed that baseline motivation was associated with OB-related quality of life at baseline and children’s initial weight [35]. Furthermore, it was found that in adults initial weight loss can positively influence change of motivation [36]. Several additional factors may potentially influence motivational changes. These include sociodemographic variables of the children and adolescents, such as age and gender, as well as parental sociodemographic characteristics, including income, migration background, and education. Potential mechanisms underlying such associations may involve differences in perceived burden or distress, as well as variations in baseline health literacy [37, 38]. Furthermore, participants’ baseline weight influences perceived burden [39] and could accordingly represent a potential predictor of motivational changes. Finally, usage data of the weight management program may be associated with motivational change, particularly given that the present weight management program incorporates motivation-enhancing methods. In turn, motivation might increase if individuals are confronted more frequently with these interventions. We therefore hypothesize that parental, children’s, and adolescents’ sociodemographic and anthropometric data, as well as baseline quality of life, an initial weight change, and the degree of engagement within the weight management program could influence a change in motivation.

To address these research gaps, we used the TTM as a theoretical framework to analyze motivational data of the large, cluster-randomized STARKIDS trial which evaluated a guideline-based multicomponent weight management program [40, 41]. The STARKIDS intervention (delivered to the intervention group (IG)) placed particular emphasis on fostering the families’ motivation and utilized techniques from Motivational Interviewing [42] as a motivation enhancing conversation strategy. We included both, data of caretakers and adolescents in the present analysis to reduce the research gap on the progress of caretakers’ motivation and to examine whether the changes in adolescents’ motivation observed in previous research [16] can be observed in the STARKIDS trial too.

The present study addressed the following research questions: [1] Does motivation regarding behavior change of caretakers and adolescents change over the course of the STARKIDS trial (pre and post data)? Do the IG (STARKIDS program) and control group (CG; optimized treatment-as-usual) differ in change of motivation over the course of the STARKIDS trial? It was hypothesized that increases on Action and Maintenance stage are higher in the IG compared to the CG in both group of parents and adolescents. [2] Which variables do predict change in motivation, respectively on active behavior change (assessed with the Action subscale) during the intervention? To address this last research question the previously described variables were used as potential predictors: age and sex of children/adolescent, caretakers’ migration background and education, family income, baseline weight and initial weight loss of children/adolescents, OB-related quality of life of children/adolescents and usage of the STARKIDS intervention.

## Methods

### Study design

This study constitutes a secondary analysis of data from the cluster-randomized STARKIDS trial [40]. The primary objective of the STARKIDS trial was to develop and evaluate a multicomponent weight management program for families with children or adolescents with OB or OW [40]. The STARKIDS study was registered in the German Clinical Trials Register (DRKS) (DRKS00022813) on 27 November 2020 which is recognized as a primary register of the World Health Organization (Universal Trial Number U1111-1254-9536). Ethical approval for this study was granted by the Ethics Committee of the Medical Faculty of the University of Tübingen (No. 620/2020BO1) and by the Ethics Committee of the Medical Association of the State of Baden-Wuerttemberg (No. B-F-2020-160).

### Participants

Pediatric and family practices in the federal state of Baden-Wuerttemberg in southern Germany (*N* = 89) were recruited. The practices were randomly allocated to IG (*n* = 44) or CG (*n* = 45). The participants of the study were children and adolescents with OB or OW, along with one of their caretakers (mostly mothers, see Table 1). The families were recruited by employees of the participating pediatric and family practices. *N* = 379 families were assigned to the IG and *n* = 232 to the CG. The inclusion criteria for children and adolescents were an age between 3 and 17 years, a body-mass-index (BMI) greater than the 90th percentile, statutory health insurance and an appropriate knowledge of German. The exclusion criteria encompassed participants who had previously engaged in an interdisciplinary weight management program for a minimum of one year, those who had undergone inpatient treatment for OW/OB within the past year, and individuals with severe other systemic diseases or severe other psycho-social stress or diseases not associated with OW/OB. For the present analysis, two subsamples were derived from the STARKIDS cohort: (a) adolescents, aged 12 years and older, and (b) caretakers (mostly mothers) of the full age range of children/adolescents (3–17 years).

### Procedure

**Fig. 2:**
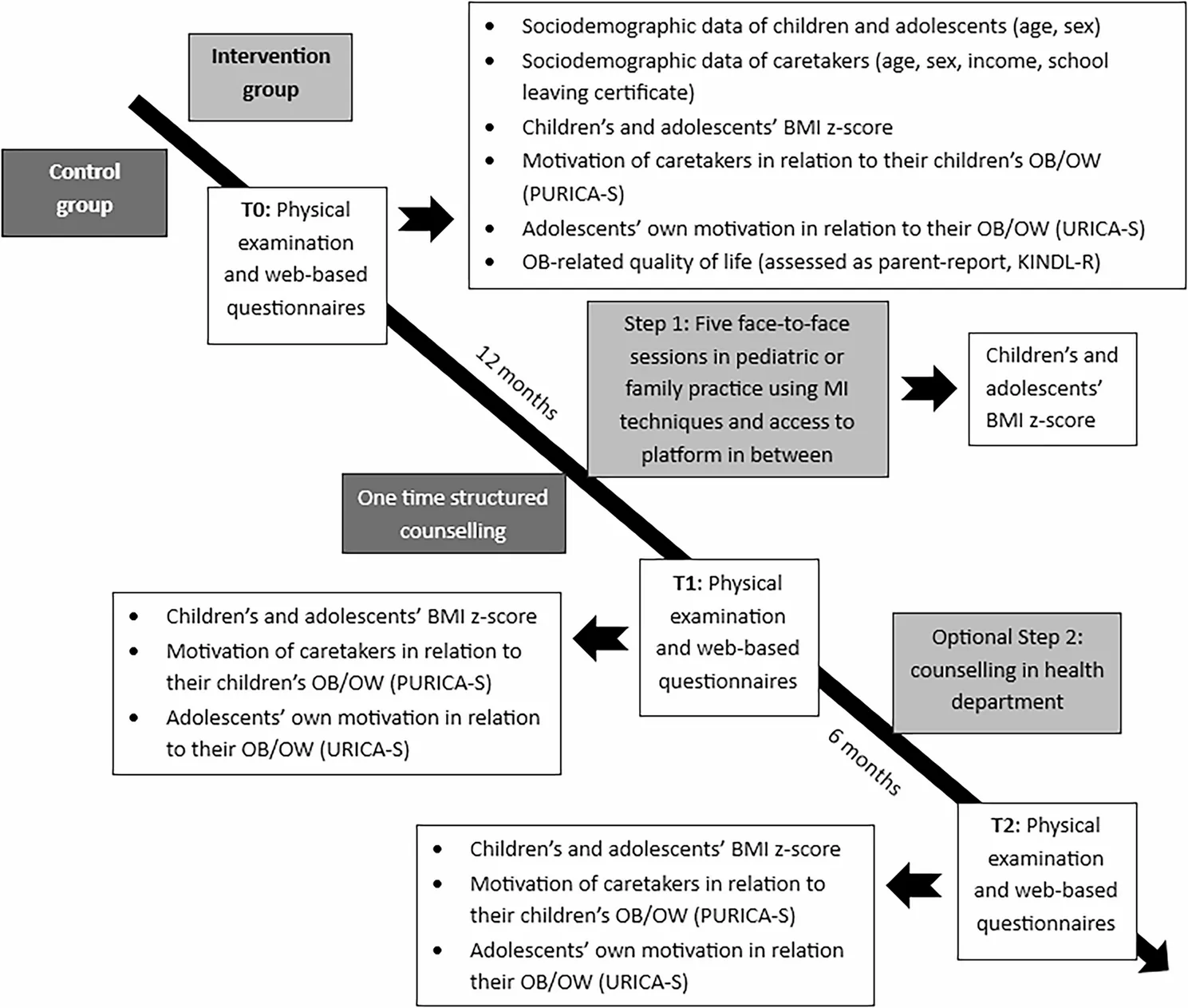
Study course of the STARKIDS trial

After recruiting and informed consent, families received an account for the STARKIDS platform. A physical examination was conducted in the pediatric or family practice and caretakers filled out a web-based questionnaire with demographic data and questionnaires (T0). Adolescents (aged 12 and older) could optionally complete a web-based questionnaire. After baseline measures, the IG received the STARKIDS program which consisted of five face-to-face sessions (called: STARKIDS Start, Eating & Drinking, Activity & Media, Family life, STARKIDS Keep on) in the pediatric or family practice and an online platform for the time in between the face-to-face sessions (step 1 of the intervention). STARKIDS was a family-focused intervention, children/adolescents were therefore expected to attend the face-to-face sessions together with at least one parent. The intervention aimed to change the families’ behaviors and aspects of their surroundings that were within the family’s own sphere of influence (e.g., eating behavior, physical activity, media usage) as well as psychological constructs within the family. A detailed description of the intervention can be found in the study protocol [40] and the main outcome paper [41]. The CG received treatment-as-usual which was a one-time structured counselling in the pediatric or family practice. After one year, both groups had another physical examination and completed the web-based questionnaire (T1). Participants of the IG with insufficient success received further counselling at the local public health department (step 2 of the intervention). After another six months, both groups completed a follow-up which again consisted of a physical examination and a questionnaire (T2). An overview of the study course is depicted in Fig. 2. The STARKIDS program was grounded in the TTM and Motivational Interviewing. The medical professionals responsible for conducting the face-to-face sessions received training in the principles and techniques of motivational interviewing, and all sessions were designed and delivered accordingly. Additionally, before every face-to-face session, caretaker (and optionally adolescents) completed a motivation questionnaire. During the session, families were presented with their scores on each SoC of the TTM which were subsequently discussed with the medical professional with a focus on barriers to and motivators for continued participation in the STARKIDS program. Further details regarding the STARKIDS study design and procedures can be gained in the study protocol [40] and in the main outcomes paper [41].

### Measures

#### Sociodemographic and anthropometric data

Sociodemographic data of caretakers, adolescents, and children were assessed in the pediatric or family practice and as part of the web-based questionnaire. Relevant for the current analysis were age and sex of children and adolescents, as well as families’ household income, caretakers’ migration background (whether in all the data for each person there was some indication for migration background) and education level (operationalized as school leaving certificate). Children’s and adolescents’ weight and height were assessed by the medical staff at all measurement time points. BMI z-scores were calculated thereof.

#### Motivation regarding behavior change

To operationalize motivation, the TTM was used as a theoretical framework. The SoC were assessed with the German short versions of the University of Rhode Island Change Assessment (URICA, [43]). Adolescents completed the URICA-S [30] scale and parents the PURICA-S [44] which measures parents SoC in relation to a health issue of their child. Caretakers and adolescents were instructed rating the items in relation to the children’s / adolescents’ OB or OW. Both questionnaires consist of 16 items, whereof four belong to each subscale (SoC): precontemplation, contemplation, action and maintenance. Descriptions of the scales are given in the introduction. Examples of the URICA-S scales are: “I’m not the problem one. It doesn’t make much sense for me to be here.” (Precontemplation), “I wish I had more ideas on how to solve the problem.” (Contemplation), “I am really working hard to change.” (Action), and “I’m here to prevent myself from having a relapse of my problem.” (Maintenance). All scales are used in the present study. Each item is answered on a 5-point Likert scale (0 = “not applicable at all”; 4 = “very strongly applicable”). Reliability and validity were shown to be adequate in previous studies [30, 44].

#### OB-related quality of life

OB-related quality of life was assessed with the OB module of the German questionnaire KINDL^R^ [45–47], for which age-appropriate versions are available for younger and older children and adolescents, as well as for their parents. The OB module consists of 15 items about possible consequences on quality of life resulting from OB/OW. Items were answered on a 5-point Likert scale (1 = “never”, 5 = “all the time”). Out of the three scaling approaches available for the KINDL^R^, the conventional total sum score scaled to a range of 0–100 was applied. All data used in the present analysis were based on parent proxy reports of their child’s or adolescent’s OB-related quality of life. Reliability and validity were good in previous studies [45–48].

#### Usage data and initial weight loss

Engagement with the STARKIDS intervention was operationalized using two variables: the number of face-to-face sessions attended (maximum five) and the number of logins to the online platform which families used between the face-to-face sessions. Both variables were recorded automatically by the STARKIDS software. Additionally, initial weight loss (operationalized as the difference in BMI z-score between the first (study begin) and the second (after three months) face-to-face sessions) was included as a predictor variable.

### Statistical analysis

All statistical analysis were conducted with the Software SPSS Version 31.0.0.0 (117). Due to a substantial number of missing values, we conducted a multiple imputation, assuming that the probability of being missing is related to other observed variables (MAR, [49]). The variables which were used as predictors in a multiple imputation (m = 500) procedure are listed in the additional file: Table 1. The multiple results are integrated according to Rubin’s rules [50]. All following inferential analyses are based on these 500 multiply imputed datasets. Descriptive data and sociodemographic data were calculated with complete cases. Level of statistical significance was set at *p* < 0.05.

Analyses were calculated separately for caretaker and for adolescents. The change of motivation was calculated with every SoC separately. Comparisons between T0 and T1 and between T0 and T2 were calculated. Linear mixed models were estimated with the dependent variable being the difference on every SoC scale between T1-T0 and T2-T0. Predictors were group (intervention or control), SoC value at t0 (centered), and interaction between these two. Random intercepts for the cluster variable Pediatric Practice were included. Effect sizes were calculated using the following formula: $\:t*\sqrt{1/n}$ as commonly used effect size measures are not directly applicable to linear mixed models.

To find predictors for the change of motivation, a multiple linear regression for the IG was calculated with the difference in the Action scale between T1-T0 being the outcome. This subscale was chosen as outcome because the STARKIDS intervention was specifically designed to target this SoC. Potential predictors were previously defined, partly based on the existing literature and comprised: children’s and adolescents’ age and sex, baseline BMI z-score, initial weight loss, caretakers’ education level, migration background, family income, OB-related quality of life, number of face-to-face sessions attended, and number of logins. All predictors were centered. Analysis are of explorative nature since previous research about associations between included variables and motivational change is limited.

## Results

**Fig. 3:**
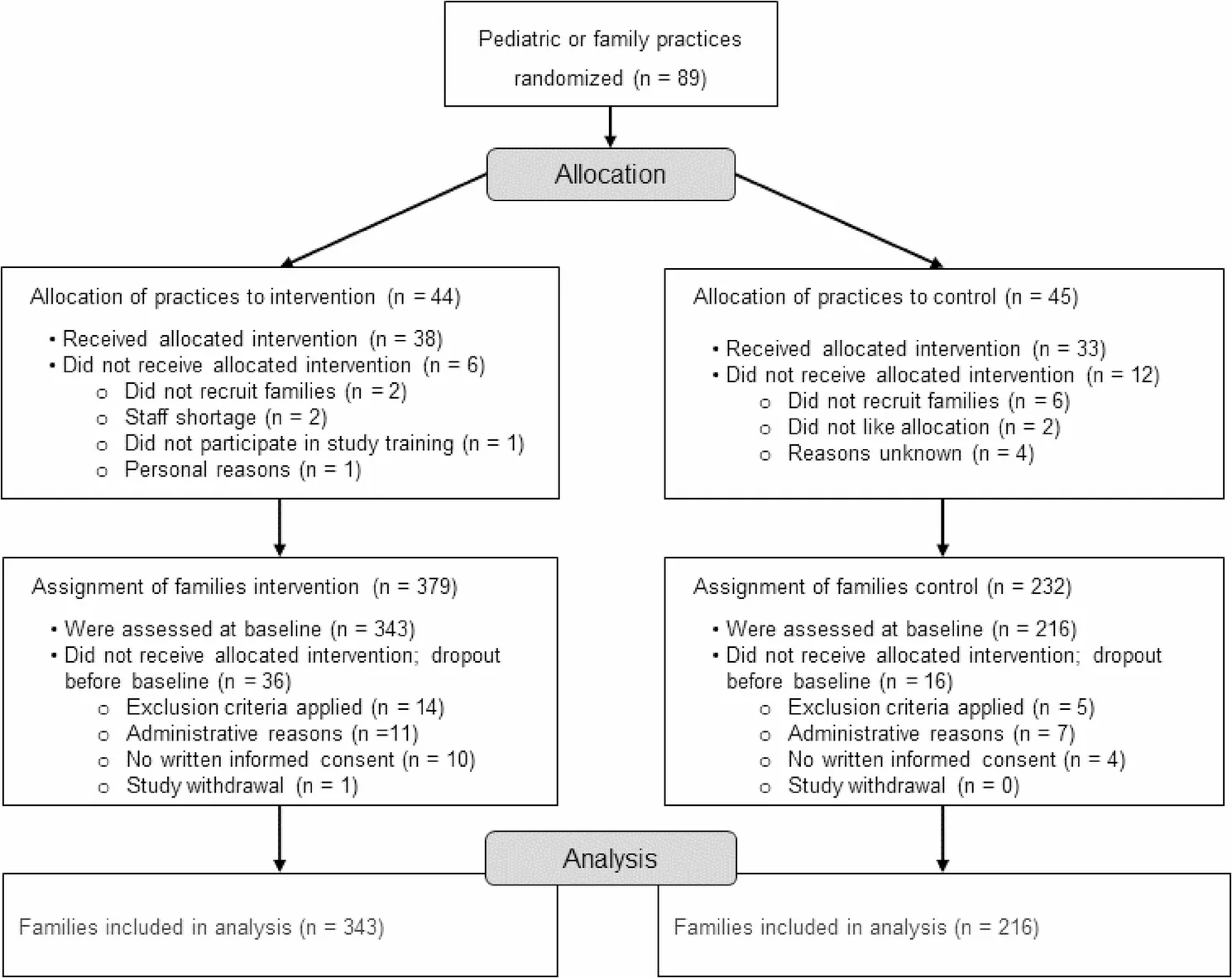
Flowchart of the study

Figure 3 displays the study flow. *N* = 343 families of the IG and *N* = 216 families of the CG were included in the analysis. Table 1 displays demographic data of study participants and further descriptive values. BMI z-score for all included children and adolescents (sample of adolescents and children of the caretakers’ sample) was 2.39 (0.50).

**Table 1:** Sociodemographic characteristics of the study participants and descriptive baseline data

	Intervention group, M (SD) or number (%)	Control group, M (SD) or number (%)
Caretakers		
Relation to adolescent (*N* = 515)		
• Mother	277 (85.5%)	175 (89.7%)
• Father	38 (11.7%)	18 (9.2%)
• Stepmother	1 (0.3%)	0
• Grandmother	5 (1.5%)	1 (0.5%)
• Adoptive mother	1 (0.3%)	1 (0.5%)
• Adoptive father	1 (0.3%)	0
• other	1 (0.3%)	0
Age in years (*N* = 506)	40.49 (6.84)	40.84 (6.72)
BMI (self-report) (*N* = 487)	29.68 (6.57)	30.06 (6.24)
Migration Background (*N* = 542)		
• yes	167 (48.7%)	85 (39.4%)
School leaving certificate (*N* = 542)		
• Lower secondary	80 (23.3%)	49 (22.7%)
• Intermediate secondary	130 (37.9%)	79 (36.6%)
• A-levels	84 (24.5%)	51 (23.6%)
• None	14 (4.1%)	4 (1.9%)
• Special education	0	2 (0.9%)
• Other	14 (4.1%)	10 (4.6%)
Household income (*N* = 542)		
• < 1000 €	17 (5.0%)	10 (4.6%)
• 1000–1999 €	67 (19.5%)	23 (10.6%)
• 2000–2999 €	86 (25.1%)	58 (26.9%)
• 3000–3999 €	76 (22.2%)	46 (21.3%)
• 4000–4999 €	53 (15.5%)	39 (18.1%)
• > 5000 €	25 (7.3%)	17 (7.9%)
KINDL^R^ obesity module	70.20 (17.55)	72.29 (17.12)
Usage: number of logins (only IG, *N* = 331)	33.76 (27.12)	n/a
Usage: number of face-to-face sessions (only IG, *N* = 331)	3.87 (1.67)	n/a
Initial weight loss in BMI z-score (only IG, *N* = 278)	0.06 (0.23)	n/a
**Adolescents**		
Age in years (*N* = 143)	13.95 (1.40)	14.18 (1.57)
Sex (*N* = 143)		
• female	29 (31.2%)	26 (48.1%)
BMI z-score (*N* = 143)	2.37 (0.43)	2.37 (0.54)

Abbreviations: BMI = body mass index; IG = intervention group; KINDL^R^ = Revidierter Fragebogen für Kinder und Jugendliche zur Erfassung der gesundheitsbezogenen Lebensqualität

### Change of motivation regarding behavior change (Pre-Post data in IG and CG)

**Fig. 4:**
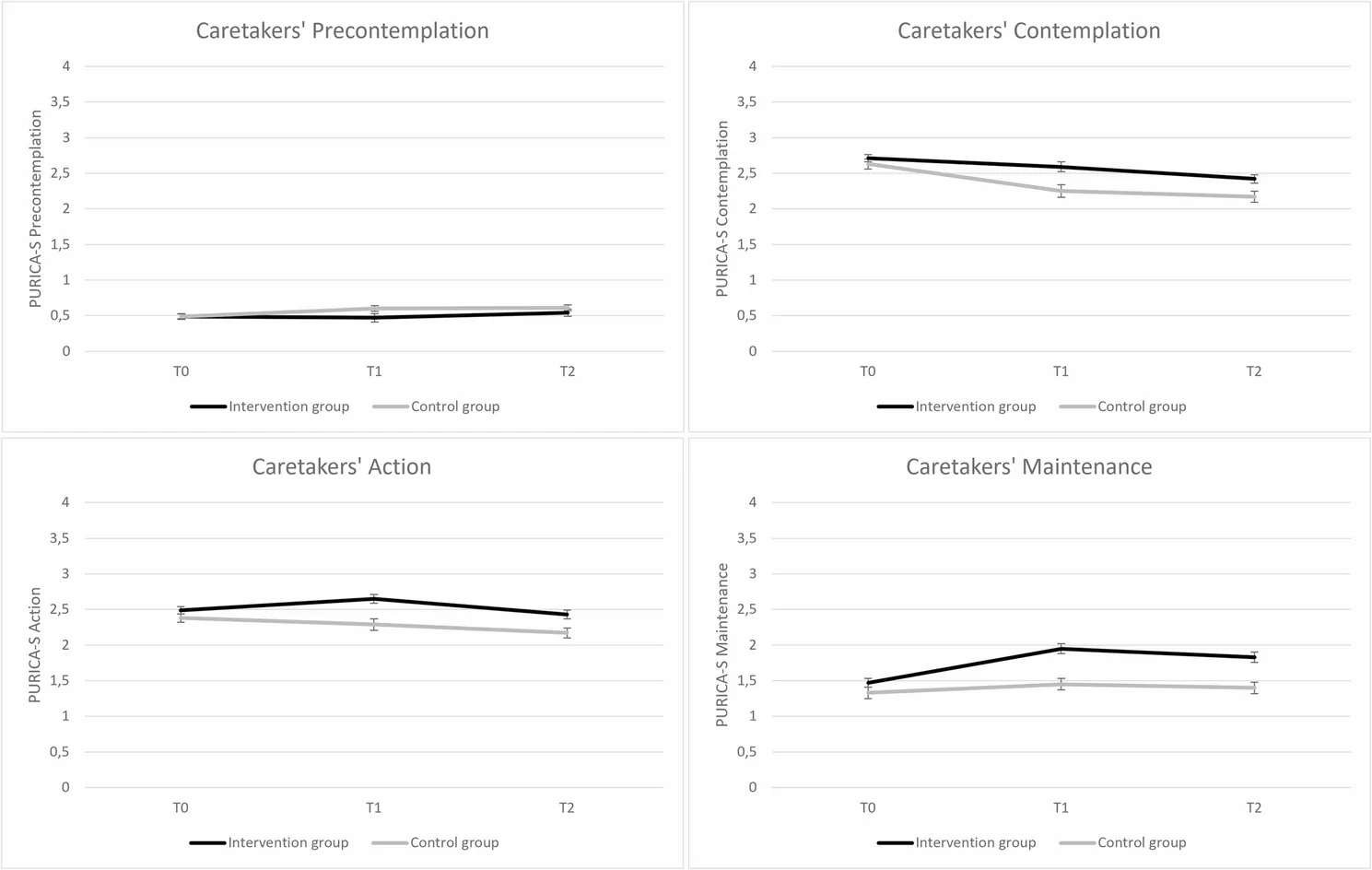
Means and standard errors of the PURICA-S subscales of caretakers at the three timepoints

Figure 4 shows the trajectories of all four TTM subscales (descriptive data) in caretakers. On the Action subscale, a descriptive increase from T0 to T1 was observed in the IG, whereas the control group showed a descriptive decrease over the same period. On the Maintenance subscale, the increase from T0 to T1 was descriptively more pronounced in the IG. Inferential statistics show that in caretakers, groups differed significantly in their change from T0 to T1 on the Maintenance subscale (t = -2.44, *p* = 0.015, effect size = -0.10). The changes from T0 to T1 on the Action subscale (t = -1.82, *p* = 0.069, effect size = -0.08) are non-significant, but warrant further investigation. No significant between-group differences were found for changes from T0 to T1 on the Precontemplation and Contemplation. Similarly, groups differed significantly in their change from T0 to T2 in the Maintenance subscale (t = -3.13, *p* = 0.002, effect size = -0.05), while no significant differences were found on the remaining subscales, whereas results for the Action subscale (t = -1.66, *p* = 0.097, effect size = -0.07) are non-significant but warrant further investigation. Descriptive values of the scales at the three timepoints, as well as test statistics are depicted in Table 2. Complete results of the linear mixed models are depicted in the additional file: Table 2 and additional file: Table 3.

**Table 2:** Descriptive values and results of linear mixed models of the PURICA-S scales (caretakers)

Scale / SoC	Intervention group, M (SD)			Control group, M (SD)			Test statistic for Group variable^a^
	T0	T1	T2	T0	T1	T2	
Pre-contemplation	0.49 (0.58) *N* = 322	0.47 (0.62) *N* = 195	0.54 (0.59) *N* = 238	0.49 (0.50) *N* = 195	0.60 (0.66) *N* = 142	0.61 (0.60) *N* = 163	T0-T1: estimate = 0.11, SE = 0.10, t = 1.19, *p* = 0.236, effect size = 0.05 T0-T2: estimate = 0.05, SE = 0.07, t = 0.63, *p* = 0.532, effect size = 0.03
Contemplation	2.71 (0.96) *N* = 323	2.59 (0.92) *N* = 195	2.42 (0.97) *N* = 237	2.62 (0.96) *N* = 194	2.25 (1.05) *N* = 145	2.17 (1.07) *N* = 162	T0-T1: estimate = -0.17, SE = 0.13, t = -1.33, *p* = 0.184, effect size = -0.06 T0-T2: estimate = -0.13, SE = 0.11, t = -1.19, *p* = 0.233, effect size = -0.05
Action	2.49 (0.90) *N* = 323	2.65 (0.86) *N* = 195	2.43 (0.86) *N* = 237	2.387(0.88) *N* = 195	2.29 (0.91) *N* = 145	2.17 (0.91) *N* = 163	T0-T1: estimate: -0.22, SE = 0.12, t = -1.82, *p* = 0.069, effect size = -0.08 T0-T2: estimate = -0.17, SE = 0.10, t = -1.66, *p* = 0.097, effect size = -0.07
Maintenance	1.47 (1.08) *N* = 322	1.95 (1.02) *N* = 194	1.83 (1.04) *N* = 237	1.33 (1.05) *N* = 192	1.45 (1.02) *N* = 144	1.40 (1.03) *N* = 162	T0-T1: estimate = -0.34, SE = 0.14, t = -2.44, *p* = 0.015, effect size = -0.10 T0-T2: estimate = -0.37, SE = 0.12, t = -3.13, *p* = 0.002, effect size = -0.05

Notes: a) Linear mixed models were calculated with the following predictors: T0 value, Group, Interaction Group x T0 value. In this column only the results of the predictor Group are displayed since it is relevant to the research question. Further results are depicted in the additional file: Table 2 and additional file: Table 3

In the adolescent sample, no significant between-group differences were observed for changes from either T0 to T1 or T0 to T2 on any subscale. Descriptive and inferential statistics are presented in Table 3. Complete results of the linear mixed models, including a descriptive graphical overview, are provided in the additional file: Table 4 and additional file: Table 5 and the additional file: Fig. 1.

**Table 3:** Descriptive values and results of linear mixed models of the URICA-S scales (adolescents)

Scale / SoC	Intervention group, M (SD)			Control group, M (SD)			Test statistic for Group variable^a^
	T0	T1	T2	T0	T1	T2	
Pre-contemplation	0.90 (0.73) *N* = 82	0.64 (0.68) *N* = 50	0.83 (0.81) *N* = 58	0.81 (0.62) *N* = 46	0.77 (0.53) *N* = 34	0.93 (0.82) *N* = 38	T0-T1: estimate = 0.02 SE = 0.08, t = 0.27, *p* = 0.784, effect size = 0.01 T0-T2: estimate = 0.02, SE = 0.09, t = 0.21, *p* = 0.836, effect size = 0.01
Contemplation	2.62 (1.02) *N* = 82	2.43 (0.96) *N* = 50	2.17 (0.99) *N* = 58	2.24 (0.95) *N* = 46	1.93 (0.88) *N* = 34	1.72 (0.99) *N* = 38	T0-T1: estimate = -0.06, SE = 0.11, t = -0.57, *p* = 0.571, effect size = -0.02 T0-T2: estimate = -0.07, SE = 0.12, t = -0.59, *p* = 0.553, effect size = -0.03
Action	2.19 (0.90) *N* = 83	2.46 (0.92) *N* = 50	2.22 (0.84) *N* = 58	2.08 (0.72) *N* = 46	1.88 (0.87) *N* = 34	1.78 (0.92) *N* = 38	T0-T1: estimate = -0.09, SE = 0.11, t = -0.80, *p* = 0.421, effect size = -0.03 T0-T2: estimate = -0.08, SE = 0.11, t = -0.76, *p* = 0.446, effect size = -0.03
Maintenance	1.69 (1.08) *N* = 81	1.86 (1.10) *N* = 50	1.73 (1.17) *N* = 58	1.40 (1.11) *N* = 46	1.32 (0.87) *N* = 34	1.27 (0.98) *N* = 38	T0-T1: estimate = -0.07, SE = 0.12, t = -0.62, *p* = 0.536, effect size = -0.03 T0-T2: estimate = -0.7, SE = 0.12, t = 0.55, *p* = 0.582, effect size = -0.02

Notes: a) Linear mixed models were calculated with the following predictors: T0 value, Group, Interaction Group x T0 value. In this column only the results of the predictor Group are displayed since it is relevant to the research question. Further results are depicted in the additional file: Table 4 and additional file: Table 4

### Predictors for the change in action scale in the IG

The multiple linear regression analysis for caretakers in the IG showed that OB-related quality of life at baseline predicted the change in the Action scale between T0 and T1 (b = -0.01, *p* < 0.001). The poorer the OB-related quality of life of children/adolescents (proxy-reported by caretakers), the larger was the increase of caretakers’ Action score between T0 and T1. Predictions by all other variables were not significantly different from 0. Results of the regression analysis are depicted in Table 4.

**Table 4:** Multiple linear regression analysis with the Action Subscale T0-T1 as outcome

	b 95% [CI]	SE	T	*p*
Intercept	-0.03 [-0.50; 0.44]	0.24	-0.13	0.900
Age of child/adolescent	0.01 [-0.04; 0.05]	0.02	0.35	0.727
Sex of child/adolescent	0.15 [-0.11; 0.41]	0.13	1.13	0.257
School leaving degree of caretaker (reference group: lower secondary)				
• Intermediate secondary	-0.05 [-0.37; 0.27]	0.16	-0.33	0.742
• A-levels	0.07 [-0.30; 0.44]	0.19	0.39	0.695
• None	-0.21 [-0.97; 0.52]	0.38	-0.58	0.560
• Special education	n/a	n/a	n/a	n/a
• Other	n/a	n/a	n/a	n/a
Migration background of caretaker	-0.05 [-0.31; 0.20]	0.13	-0.41	0.680
Household income (reference group: < 1000 €)				
• 1000–1999 €	-0.18 [-0.66; 0.29]	0.24	-0.76	0.448
• 2000–2999 €	-0.23 [-0.69; 0.23]	0.23	-0.97	0.334
• 3000–3999 €	-0.06 [-0.52; 0.41]	0.24	-0.24	0.813
• 4000–4999 €	-0.10 [-0.58, 0.39]	0.25	-0.38	0.703
• > 5000 €	n/a	n/a	n/a	n/a
BMI z-score of child/adolescent at T0	0.02 [-0.26; 0.31]	0.14	0.17	0.868
Initial weight loss	0.11 [-0.45; 0.66]	0.28	0.37	0.709
Parent-reported OB-related quality of life	-0.01 [-0.02; -0.01]	0.00	-3.42	**< 0.001**
Usage: number of face-to-face sessions attended	0.02 [-0.09; 0.12]	0.05	0.32	0.749
Usage: number of logins	-0.00 [-0.01; 0.00]	0.00	-1.10	0.286

Notes: Occasionally levels of categorical variables were not present in the sample. Abbreviations: BMI = body mass index, OB = obesity

## Discussion

The present study investigated longitudinal changes in motivation regarding behavior change among adolescents and caretakers participating in the STARKIDS weight management program, using the TTM as an operationalizing framework. Among caretakers, participation in the motivation-focused intervention was associated with significantly greater motivational change on the Maintenance subscale compared to the control group, both from T0 to T1 and from T0 to T2 as well as (non-significant) trends on the Action subscale. No significant between-group differences in motivational change were observed in the adolescent sample. Multiple linear regression analysis further identified OB-related quality of life at baseline as the only significant predictor of change on the Action subscale from T0 to T1 among caretakers in the IG.

The descriptive data revealed consistently low Precontemplation scores across both groups and all time points, which is broadly consistent with the expectation that families who voluntarily enroll in a weight management program are already aware of the need for behavioral change. Conversely, higher scores on the Contemplation, Action, and Maintenance subscales at baseline likely reflect prior attempts at weight management before study entry. The comparatively lower Maintenance scores at T0 relative to the Action scale suggest that families had begun engaging in weight management behaviors but had not yet consolidated these sufficiently to focus on relapse prevention, which is the behavioral domain captured by the Maintenance subscale. This lower baseline level may have created greater capacity for change on the Maintenance subscale, potentially contributing to the observed between-group differences at this stage while only a trend emerged on the Action subscale. It is noteworthy that Action scores increased descriptively in the IG from T0 to T1 while decreasing in the control group, and the corresponding p-value approached conventional significance thresholds. This pattern suggests that the STARKIDS intervention may also have positively influenced motivation at the Action stage, though this effect did not reach statistical significance in the present analysis. High values on the Action stage may reflect families’ problem awareness of the need for behavior change and their active engagement into adopting healthier behaviors. The observed increase in Action scores within the IG suggests that families successfully implemented what they have learned from the face-to-face sessions and the online platform into behavioral change. Although this interpretation should be considered tentative given that the between-group difference on this subscale did not quite reach statistical significance.

Since parents and caretakers act as “agents of change” [23] and play an important role in pediatric weight management [20, 21], their motivation is of comparable clinical relevance to that of affected children and adolescents. Since children cannot conduct behavior changes necessary for weight management (e.g. cooking habits) on their own, parental involvement is required. Therefore, parental motivation needs to be given more attention. Furthermore, given that multiple family members are frequently affected by OW or OB [51], as was also observed in the present sample, modifying the lifestyle patterns of entire families may yield more extensive benefits. In addition, parental motivation is especially important in our study since in the sample of adolescents, no significant differences in any of the SoC between T0 and T1, as well as between T0 and T2 were observed. This is contrary to previous research: a systematic review [16] found an increase in adolescents’ motivation in IGs of different studies. One possible reason therefore could be heterogenous measures and study designs described in the review which are hardly comparable. Several explanations may account for the absence of an intervention effect among adolescents in the present analysis. First, the sample of adolescents was smaller than the sample of caretakers, which may have resulted in reduced statistical power and limited the ability to detect potential effects. Second, motivational processes may differ between caretakers and their offspring. During adolescence, peers become increasingly important influences on motivation and health-related behaviors [52]. However, the STARKIDS intervention primarily addressed adolescents within their family context and did not explicitly incorporate peer-based components. Moreover, the broad age range of 12 to 17 years includes adolescents at different developmental stages, which may have introduced considerable heterogeneity and potentially obscured intervention effects. Third, caregivers’ motivation may be more responsive to a motivation-focused intervention than adolescents’ motivation due to differences in cognitive maturity and because of more behavioral opportunities to actively change behavior. Caregivers typically have greater opportunities to actively implement lifestyle changes, as they are mostly responsible for food purchasing, meal preparation, and structuring family environment. In contrast, adolescents remain more dependent on their caregivers and may have fewer possibilities to translate motivation into behavioral change. This is in line with previous findings that pediatric weight management programs led to larger effects when parents are actively involved [53].

A significant between-group difference in motivational change on the Maintenance subscale was also observed over the longer interval from T0 to T2, a finding that is consistent with the overall structure of the STARKIDS trial (see Fig. 2). During the period from T0 to T1, the IG received the motivation-focused weight management program, which primarily targeted active behavioral change, reflected in the Action subscale of the TTM. When step 1 of the intervention was completed (after 12 months), families in the IG with insufficient success transferred to step 2, however, as reported elsewhere [41] this opportunity was rarely utilized, meaning that for the majority of families, active program participation ended at T1. Therefore, in the six-month period between T1 and T2, families no longer received a structured support and were instead required to consolidate and maintain the behavioral changes so far. It therefore seems reasonable that over the longer T0-T2 time period a significant between-group difference emerged specifically on the Maintenance subscale of the PURICA-S reflecting families’ efforts to sustain what they have learned. This finding is of particular clinical relevance since behavior changes for weight management need to be implemented in daily lives in the long run, otherwise a re-gain of weight is likely. Higher levels of motivation at the Maintenance stage may serve as a protective factor supporting families in this critical phase.

Among the ten candidate predictors examined for the second research question, OB-related quality of life at baseline emerged as the sole significant predictor of change on the Action subscale from T0 to T1 for caretakers in the IG. Specifically, lower OB-related quality of life at baseline was associated with greater increases in Action scores over this period, indicating that caretakers of children experiencing a higher burden from the weight of their child showed the greatest motivational gains. This finding is in line with previous research on the same trial [35]: Baseline motivation was similarly associated with OB-related quality of life in both adolescents’ self-report and parental proxy-report. OB-related quality of life therefore seems to be a particularly relevant correlate of motivation in pediatric weight management. While associations between quality of life, motivation and weight loss have been reported previously in adolescents [54, 55], so far, no studies have reported on relations to this specific OB-related quality of life. From a clinical perspective, these findings suggest that the perceived burden of OB-related impairments may itself serve as a motivational resource that health professionals could leverage, for instance through Motivational Interviewing [42], to strengthen engagement with weight management programs. Other explanations may account for the finding of OB-related quality of life being a predictor for motivational improvement: Children and adolescents with OB and OW generally report lower levels of quality of life than their normal-weight peers [39]. Lower baseline quality of life, or more broadly reduced well-being, may be associated with lower baseline levels of motivation [56, 57], thereby providing greater potential for motivational improvement, as observed in the present analysis. However, previous research has more often reported the opposite pattern, suggesting that lower baseline quality of life may be associated with higher baseline motivation to engage in weight management [35, 58]. In such cases, initial treatment success may further reinforce motivation. These apparently conflicting interpretations indicate that the relationship between quality of life and motivation is likely influenced by additional psychological or contextual factors. In addition, poor quality of life at baseline may be perceived as a need for change, thereby enhancing readiness to engage in treatment which in turn can lead to a motivational increase. Nevertheless, it should be noted that causal inferences should not be drawn from the present analysis.

Next to OB-related quality of life no other candidate predictor differed significantly from 0. In the current sample, none of the included demographic variables predicted a change in Action scale. Therefore, in terms of a motivational gain, we did not observe a discrimination of families with specific sociodemographic variables, such as migration background, lower education or lower income. This is in contrast to other findings in OB research where families with these properties are at a disadvantage [59, 60] and should be further investigated. Furthermore, different types of variables were included in the multiple regression and have been treated equally in the model. However, if they had reached significance, the variables differed in their potential interpretability and clinical implications. Sociodemographic predictors for a motivational increase could provide valuable information about groups at risk for a lack of motivational improvement, but would offer limited opportunities for intervention, as demographic characteristics are generally not modifiable. In contrast, significant associations between psychosocial variables (as OB-related quality of life) or program-specific variables (as number of attended sessions and number of logins) and motivational increase, could potentially indicate modifiable treatment targets. Accordingly, interventions delivered prior to or during weight management could aim to improve quality of life or to improve circumstances to facilitate a more frequent participation. However, measures of adherence (here number of attended sessions and logins) may themselves reflect a certain degree of motivation and could potentially act as mediator rather than predictors. This may partly explain why these variables did not reach statistical significance when included as predictors of motivational improvement.

The present analyses were conducted within the framework of the TTM, with increases on Action and Maintenance stage interpreted as an indicator of more active behavior change. In addition to the TTM, the SDT is widely applied in pediatric weight management interventions [16] and provides a complementary framework for understanding the mechanisms underlying the STARKIDS intervention. The SDT names the three basic psychological needs for autonomy, competence, and relatedness as being essential for fostering intrinsic motivation [25]. Several components of the STARKIDS interventions, including the use of MI explicitly address these psychological needs. The classification system by Ahmadi et al. [61] lists intervention strategies linked to these needs. Some of them are implements in the STARKIDS trial: Autonomy was supported by allowing families to choose freely their desired goals in the face-to-face sessions. The usage of MI further promoted autonomous decision-making by emphasizing collaboration and minimizing external pressure. Competence was fostered through regular feedback, positive reinforcement, and reflection on progress achieved between the face-to-face sessions. Relatedness was promoted by involving the whole families as a unit and by adopting a collaborative, non-authoritarian communication style characteristic of MI. Taken together, although the STARKIDS intervention used the TTM to operationalize and assess motivation, it also incorporated key principled of the SDT. These SDT-informed components may address some of the limitations that have been raised regarding the TTM and provide additional theoretical support for the intervention’s motivational approach.

### Limitations and strengths

To the best of our knowledge, this study is among the first to examine the development of motivation regarding behavior change over the course of a motivation-focused weight management program in both adolescents and caretakers. Several limitations of the present study warrant consideration. First, the adolescent and caretaker subsamples differed in size which may have contributed to the divergent pattern of findings across the two groups. Second, the study sample predominantly consisted of families with OB, which may limit the generalizability of the findings to the broader population of children and adolescents with OW. Third, motivation respectively the scores on the SoC were assessed as self-report. This could lead to biases, especially in the field of motivation reporting, participants might have shown social desirability bias. The same applies for the use of proxy-reports for the assessment of OB-related quality of life. The TTM stages were assessed using the continuous subscale scores of the (P)URICA-S rather than as mutually exclusive categorical stages. While this scoring approach corresponds to the one originally proposed by the instrument’s developers, it does not permit a direct classification of participants into single stages, and findings should therefore be interpreted at the level of stage-related attitudes rather than discrete stage membership. Finally, the absence of a longer follow-up beyond 18 months limits conclusions regarding the sustainability of motivational change over time. Notwithstanding these limitations, there are several strengths of the current study: Its large sample size enhances statistical power and the use of multiple imputation to account for missing data reduces the risk of biased estimates. The application of the TTM as a well-established theoretical framework further enhances comparability with prior research in this field.

### Conclusions

The present findings suggest that a motivation-focused weight management program for pediatric OW and OB can produce a motivational increase at the Maintenance stage in caretakers, supporting families in consolidating behavioral changes beyond the active intervention period. Additionally, OB-related quality of life at baseline emerged as a significant predictor of motivational change, highlighting its potential as a clinically meaningful marker. Health professionals may benefit from routinely assessing this construct and integrating it into motivational counseling strategies to tailor intervention approaches accordingly. Future research may build on these findings by identifying distinct motivational trajectories or subgroups, as different patterns of motivational change may be associated with differential treatment outcomes and could inform more individualized approaches to pediatric weight management.

## Supplementary information

Below is the link to the electronic supplementary material.

Supplementary Material 1

## Data Availability

The datasets analyzed during the current study and the statistical code are available from the corresponding author upon reasonable request.

## Abbreviations

BMI: body mass index
CG: control group
IG: intervention group
OB: obesity
OW: overweight
KINDL^R^: Revidierter Fragebogen für Kinder und Jugendliche zur Erfassung der gesundheitsbezogenen Lebensqualität (English: Revised questionnaire for children and adolescents to assess health-related quality of life)
SDT: Self-determination theory
SoC: stages of change
STARKIDS: Stufenmodell Adipositaspraevention und -therapie im Kindes- und Jugendalter (English: stepwise model for obesity prevention and treatment in children and adolescents)
TTM: Transtheoretical model (by Prochaska and DiClemente)
URICA: University of Rhode Island Change Assessment
URICA-S: University of Rhode Island Change Assessment-Short
PURICA-S: Parent Perspective University of Rhode Island Change Assessment-Short
