# Supplementary material for: Motivation in adolescents and caretakers undergoing a weight management program – longitudinal data from the cluster-randomized, motivation-focused STARKIDS trial
Source: BMC Med. 2026 Nov 4;24:621. doi: 10.1186/s12916-026-05332-7 (PMC13744128; doi:10.1186/s12916-026-05332-7)

<u>Additional file</u>

Motivation in adolescents and caretakers undergoing a weight management program – longitudinal data from the cluster-randomized, motivation-focused STARKIDS trial

Anne Herschbach, Florian Junne, Rebecca Erschens, Stefan Ehehalt, Johann Jacoby, Peter Martus, Isabelle Mack, Alisa Weiland, Inga Krauß, Constanze Greule, Gorden Sudeck, Guido Zurstiege, Annalena Wels, Sophia Helen Adam, Andreas Hoell, Wolfgang Bethge, Monika Ums, Torben Sammet, Maren Reyer, Olaf Schliesing, Stephan Zipfel, Caterina Gawrilow, Katrin Elisabeth Giel, Katrin Ziser

Content:

Additional file: table 1: Further information on the multiple imputation

Additional file: table 2: Detailed results of the mixed models for PURICA-S (caretakers) scales for the difference T0-T1 as the dependent variable

Additional file: table 3: Detailed results of the mixed models for PURICA-S (caretakers) scales for the difference T0-T2 as the dependent variable

Additional file: table 4: Detailed results of the mixed models for URICA-S (adolescents) scales for the difference T0-T1 as the dependent variable

Additional file: table 5: Detailed results of the mixed models for URICA-S (adolescents) scales for the difference T0-T2 as the dependent variable

Additional file: figure 1: Figure showing the time courses of the URICA-S scales

Additional file: table 1: Further information on the multiple imputation

SEED used in SPSS: 7332439

Variables used as predictors for the multiple imputation:

- ID of pediatric or family practice
- BMI z-score T0
- BMI z-score T1
- BMI z-score T2
- URICA-S subscale Precontemplation T0
- URICA-S subscale Contemplation T0
- URICA-S subscale Action T0
- URICA-S subscale Maintenance T0
- URICA-S subscale Precontemplation T1
- URICA-S subscale Contemplation T1
- URICA-S subscale Action T1
- URICA-S subscale Maintenance T1
- URICA-S subscale Precontemplation T2
- URICA-S subscale Contemplation T2
- URICA-S subscale Action T2
- URICA-S subscale Maintenance T2
- PURICA-S subscale Precontemplation T0
- PURICA-S subscale Contemplation T0
- PURICA-S subscale Action T0
- PURICA-S subscale Maintenance T0
- PURICA-S subscale Precontemplation T1
- PURICA-S subscale Contemplation T1
- PURICA-S subscale Action T1
- PURICA-S subscale Maintenance T1
- PURICA-S subscale Precontemplation T2
- PURICA-S subscale Contemplation T2
- PURICA-S subscale Action T2
- PURICA-S subscale Maintenance T2
- Group (intervention or control)
- Immigration to Germany of primary caretaker
- Family income
- Weight of primary caretaker
- Weight of second caretaker
- Age of child/adolescent at T0
- Sex of child/adolescent
- School leaving degree of primary caretaker
- Kindl obesity module parental report sum of 100 at T0
- Kindl obesity module parental report sum of 100 at T1
- Kindl obesity module parental report sum of 100 at T2
- BMI z-score at Start module (only intervention group)
- BMI z-score at eating and drinking module (only intervention group)
- BMI z-score at activity and media module (only intervention group)
- BMI z-score at family life module (only intervention group)
- BMI z-score at Keep On module (only intervention group)

Additional file: table 2: Detailed results of the mixed models for PURICA-S (caretakers) scales for the difference T0-T1 as the dependent variable

predictor	Estimate [CI]	SE	t	p	Effect size: $t * \sqrt{1/559}$
**Precontemplation**					
intercept	-0.08 [-0.18; 0.02]	0.05	-1.62	0.105	-0.07
T0 value	0.67 [0.54; 0.80]	0.07	10.13	<0.001	0.43
Group	0.11 [-0.08; 0.30]	0.10	1.19	0.236	0.05
Interaction Group x T0 value	0.06 [-0.19; 0.31]	0.13	0.46	0.644	0.02
**Contemplation**					
Intercept	0.24 [0.10; 0.36]	0.06	3.66	<0.001	0.15
T0 value	0.56 [0.45; 0.66]	0.05	10.74	<0.001	0.45
Group	-0.17 [-0.41; 0.08]	0.13	-1.33	0.184	-0.06
Interaction Group x T0 value	0.14 [-0.04; 0.33]	0.10	1.51	0.131	0.06
**Action**					
Intercept	-0.09 [-0.21; 0.05]	0.06	-1.38	0.168	-0.06
T0 value	0.67 [0.57; 0.78]	0.05	12.63	<0.001	0.53
Group	-0.22 [-0.46; 0.02]	0.12	-1.82	0.069	-0.08
Interaction Group x T0 value	0.13 [-0.05; 0.32]	0.10	1.40	0.161	0.06
**Maintenance**					
intercept	-0.28 [-0.42; -0.14]	0.07	-3.94	<0.001	-0.17
T0 value	0.66 [0.57; 0.76]	0.05	13.76	<0.001	0.58
Group	-0.34 [-0.60; -0.07]	0.14	-2.44	**0.015**	-0.10
Interaction Group x T0 value	0.02 [-0.16; 0.20]	0.09	0.21	0.838	0.01

Additional file: table 3: Detailed results of the mixed models for PURICA-S (caretakers) scales for the difference T0-T2 as the dependent variable

predictor	Estimate [CI]	SE	t	p	Effect size: $t * \sqrt{1/559}$
**Precontemplation**					
intercept	-0.09 [-0.17; -0.02]	0.04	-2.61	0.009	-0.11
T0 value	0.64 [0.53; 0.75]	0.06	11.32	<0.001	0.48
Group	0.05 [-0.10; 0.19]	0.07	0.63	0.532	0.03
Interaction Group x T0 value	0.13 [-0.09; 0.34]	0.11	1.17	0.241	0.05
**Contemplation**					
intercept	0.39 [0.28; 0.50]	0.06	7.12	<0.001	0.30
T0 value	0.55 [0.45; 0.65]	0.05	11.08	<0.001	0.47
Group	-0.13 [-0.34; 0.08]	0.11	-1.19	0.233	-0.05
Interaction Group x T0 value	0.12 [-0.07; 0.30]	0.10	1.25	0.213	0.05
**Action**					
intercept	0.12 [0.02; 0.22]	0.05	2.42	0.016	0.10
T0 value	0.67 [0.58; 0.76]	0.05	14.09	<0.001	0.60
Group	-0.17 [-0.36; 0.03]	0.10	-1.66	0.097	-0.07
Interaction Group x T0 value	0.06 [-0.12; 0.24]	0.10	0.65	0.517	0.03
**Maintenance**					
intercept	-0.17 [-0.29; -0.06]	0.06	-2.92	0.004	-0.12
T0 value	0.66 [0.57; 0.75]	0.05	14.48	<0.001	0.61
Group	-0.37 [-0.60; -0.14]	0.12	-3.13	**0.002**	-0.05
Interaction Group x T0 value	0.02 [-0.15; 0.19]	0.09	0.23	0.819	0.01

Additional file: table 4: Detailed results of the mixed models for URICA-S (adolescents) scales for the difference T0-T1 as the dependent variable

predictor	Estimate [CI]	SE	t	p	Effect size: $t * \sqrt{1/559}$
**Precontemplation**					
intercept	0.19 [0.11; 0.27]	0.04	4.82	<0.001	0.20
T0 value	0.99 [0.87; 1.10]	0.06	16.47	<0.001	0.70
Group	0.02 [-0.13; 0.18]	0.08	0.27	0.784	0.01
Interaction Group x T0 value	-0.01 [-0.21; 0.19]	0.10	-0.07	0.944	-0.00
**Contemplation**					
intercept	0.14 [0.03; 0.25]	0.06	2.45	0.014	0.10
T0 value	0.92 [0.78; 1.06]	0.07	12.85	<0.001	0.54
Group	-0.06 [-0.30; 0.16]	0.11	-0.57	0.571	-0.02
Interaction Group x T0 value	-0.02 [-0.23; 0.18]	0.10	-0.19	0.847	-0.01
**Action**					
intercept	-0.07 [-0.19; 0.04]	0.06	-1.20	0.213	-0.05
T0 value	0.93 [0.78; 1.1]	0.08	12.05	<0.001	0.51
Group	-0.09 [-0.31; 0.13]	0.11	-0.80	0.421	-0.03
Interaction Group x T0 value	-0.04 [-0.27; 0.20]	0.12	-0.30	0.765	-0.01
**Maintenance**					
intercept	0.04 [-0.07; 0.15]	0.06	0.68	0.499	0.03
T0 value	1.0 [0.84; 1.09]	0.06	15.02	<0.001	0.64
Group	-0.07 [-0.30; 0.16]	0.12	-0.62	0.536	-0.03
Interaction Group x T0 value	-0.01 [-0.20; 0.19]	0.10	-0.06	0.951	-0.00

Additional file: table 5: Detailed results of the mixed models for URICA-S (adolescents) scales for the difference T0-T2 as the dependent variable

predictor	Estimate [CI]	SE	t	p	Effect size: $t * \sqrt{1/559}$
**Precontemplation**					
intercept	0.02 [-0.08; 0.11]	0.05	0.32	0.746	0.01
T0 value	0.93 [0.79; 1.07]	0.07	13.10	<0.001	0.55
Group	0.02 [-0.16; 0.20]	0.09	0.21	0.836	0.01
Interaction Group x T0 value	-0.02 [-0.25; 0.21]	0.12	-0.16	0.875	-0.01
**Contemplation**					
Intercept	0.36 [0.25; 0.48]	0.06	6.02	<0.001	0.25
T0 value	0.94 [0.80; 1.08]	0.07	13.14	<0.001	0.56
Group	-0.07 [-0.31; 0.17]	0.12	-0.59	0.553	-0.03
Interaction Group x T0 value	-0.02 [-0.22; 0.19]	0.10	-0.15	0.884	-0.01
**Action**					
Intercept	0.10 [0.00; 0.21]	0.06	1.90	0.058	0.08
T0 value	0.96 [0.78; 1.14]	0.09	10.36	<0.001	0.44
Group	-0.08 [-0.30; 0.13]	0.11	-0.76	0.446	-0.03
Interaction Group x T0 value	0.01 [-0.23; 0.25]	0.12	0.10	0.925	0.00
**Maintenance**					
Intercept	0.11 [-0.02; 0.24]	0.07	1.73	0.083	0.07
T0 value	0.89 [0.76; 1.02]	0.07	12.96	<0.001	0.55
Group	-0.07 [-0.31; 0.17]	0.12	-0.55	0.582	-0.02
Interaction Group x T0 value	-0.00 [-0.20; 0.19]	0.10	-0.03	0.980	-0.00

Additional file: figure 1: Figure showing the time courses of the URICA-S scales

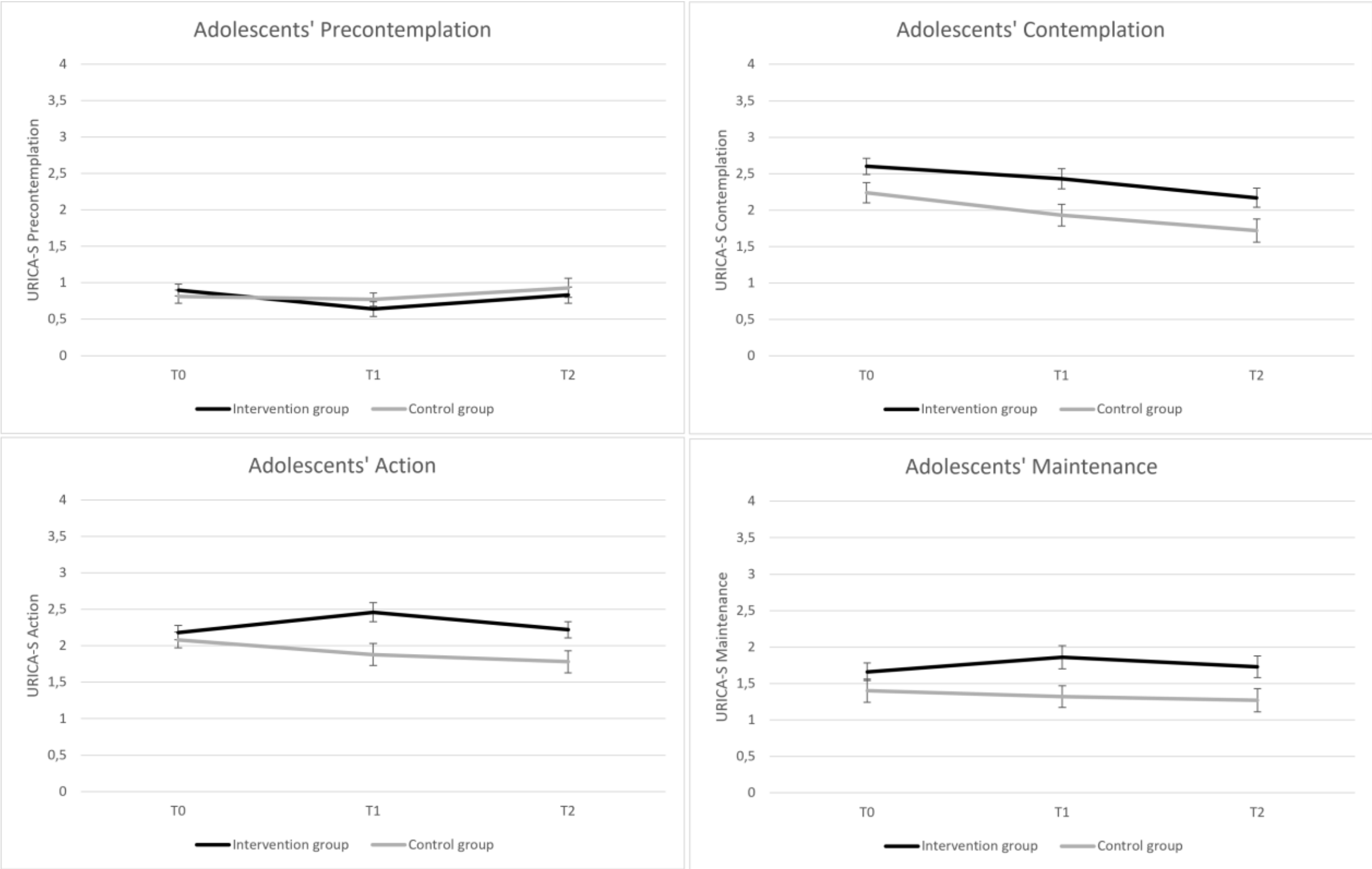

Supplement: Supplementary file 1 [file 12916_2026_5332_MOESM1_ESM.pdf]
